# Supplementary figures and images for: Foliar Symptomology, Nutrient Content, Yield, and Secondary Metabolite Variability of Cannabis Grown Hydroponically with Different Single-Element Nutrient Deficiencies
Source: Plants (Basel). 2023 Jan 17;12(3):422. doi: 10.3390/plants12030422 (PMC9920212; doi:10.3390/plants12030422)

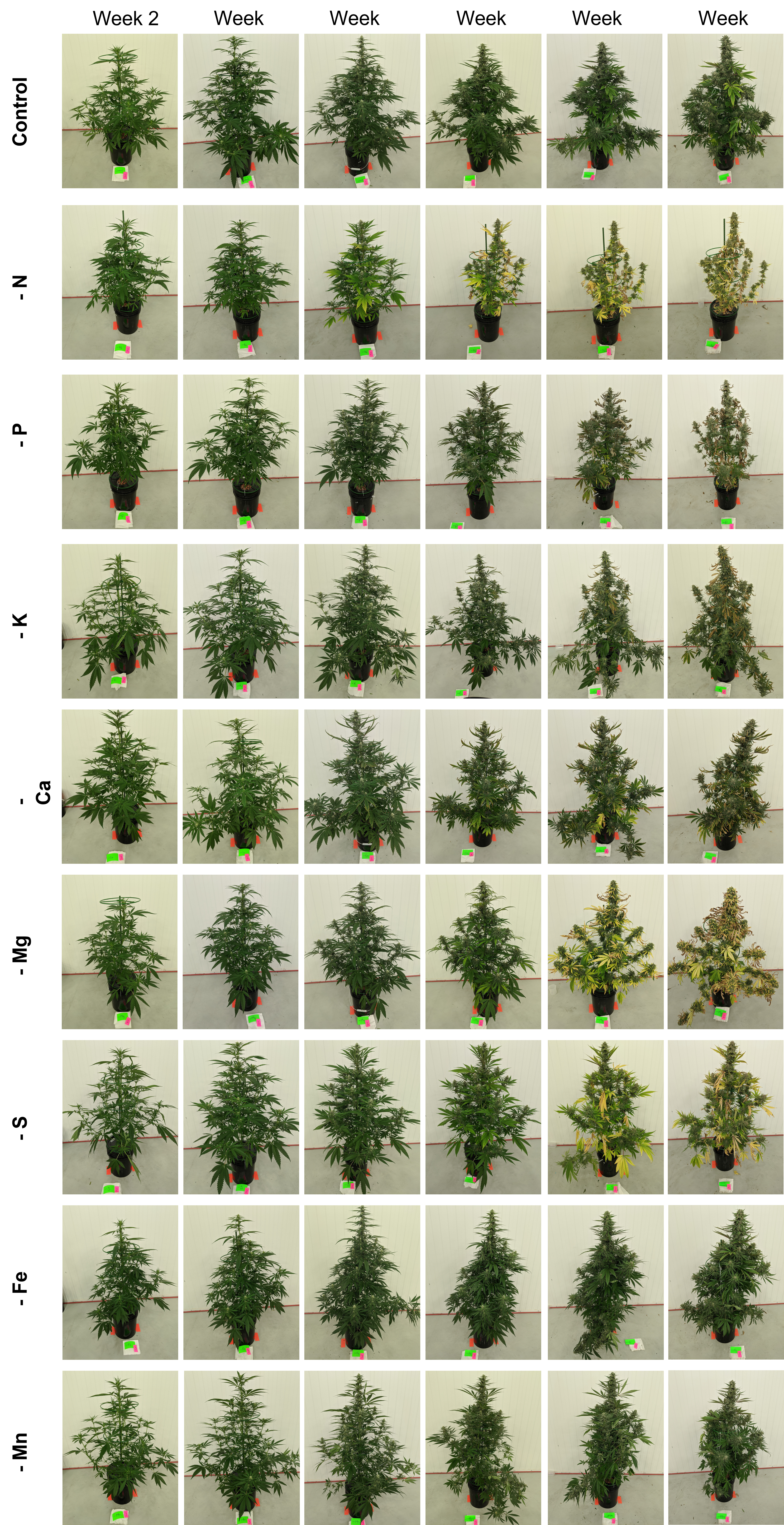

Supplement: Supplementary file 1 [file plants-12-00422-s001.zip › plants-2101397/Figure S1.png]

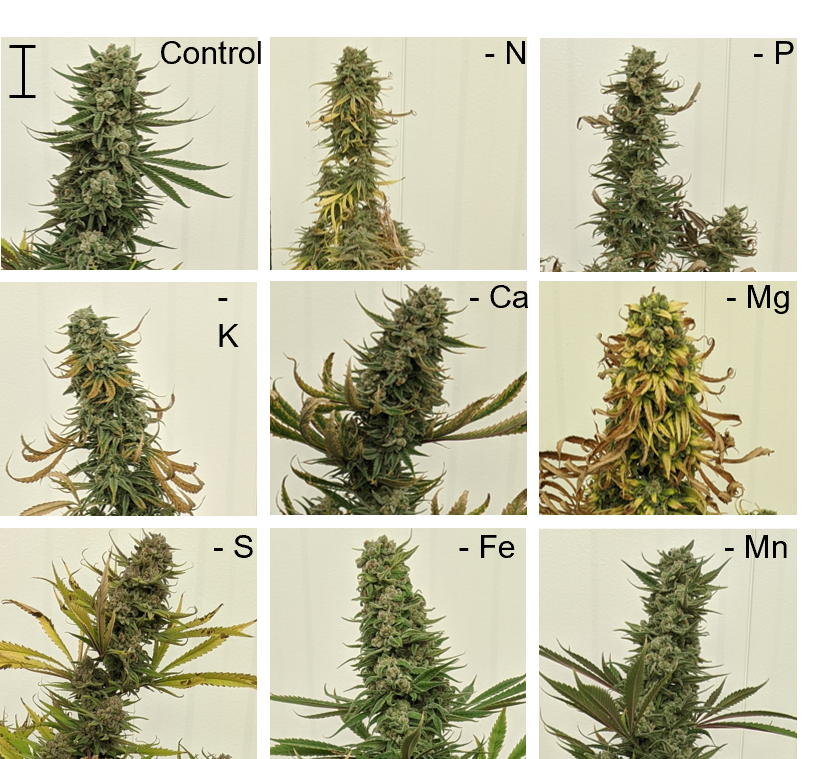

Supplement: Supplementary file 1 [file plants-12-00422-s001.zip › plants-2101397/Figure S2.png]

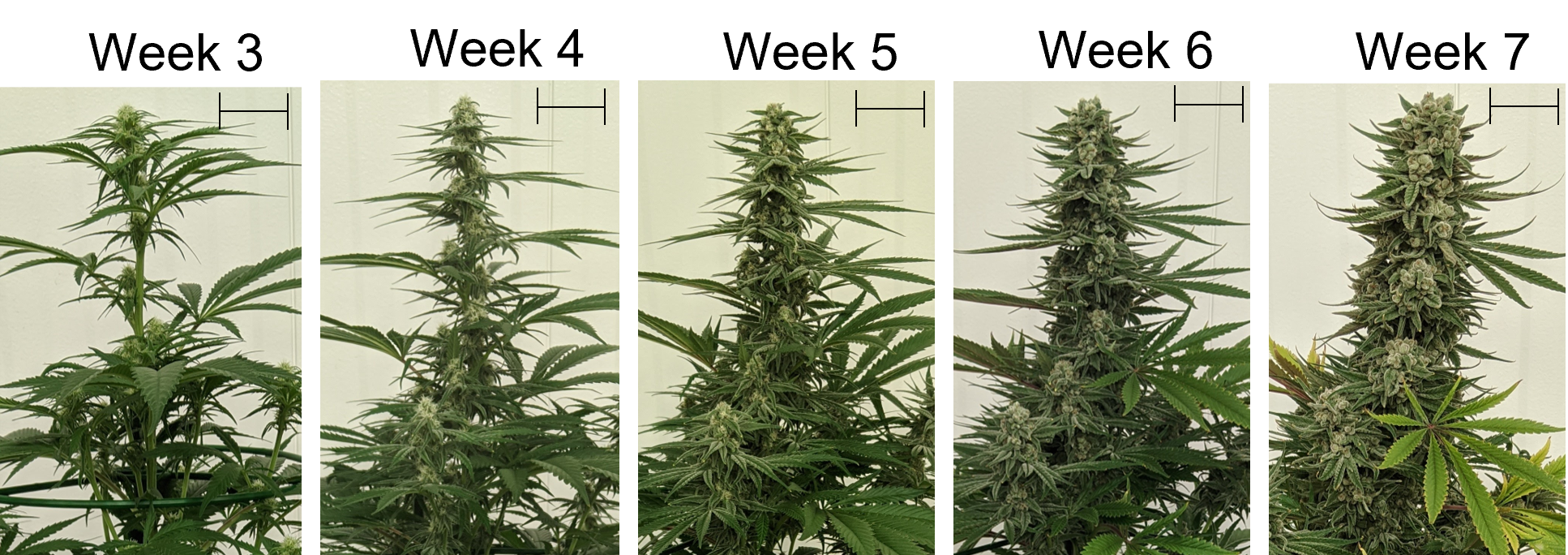

Supplement: Supplementary file 1 [file plants-12-00422-s001.zip › plants-2101397/Figure S3.png]
